# Sexually selected male weapon is associated with lower inbreeding load but higher sex load in the bulb mite

**DOI:** 10.1111/evo.14033

**Published:** 2020-07-06

**Authors:** Aleksandra Łukasiewicz, Małgorzata Niśkiewicz, Jacek Radwan

**Affiliations:** ^1^ Evolutionary Biology Group Adam Mickiewicz University Poznan Poland

**Keywords:** Alternative reproductive tactics, good genes, male weapons, sexual conflict, sexual selection, sexually antagonistic selection

## Abstract

Elaborate sexually selected ornaments and armaments are costly but increase the reproductive success of their bearers (usually males). It has been postulated that high‐quality males can invest disproportionately more in such traits, making those traits honest signals of genetic quality. However, genes associated with such traits may have sexually antagonistic effects on fitness. Here, using a bulb mite *Rhizoglyphus robini*, a species in which a distinct dimorphism exists between males in the expression of a sexually selected weapon, we compare inbreeding and gender load between lines derived from armed fighters and unarmed scramblers. After four generations of sib‐mating, inbreeding depression for female fitness was significantly lower in fighter‐derived lines compared to scrambler‐derived lines, suggesting that fighter males had significantly higher genetic quality. However, outbred females from fighter‐derived lines had significantly lower fitness compared to outbred females from scrambler‐derived lines, demonstrating significant gender load associated with the presence of a sexually selected male weapon. Our results imply that under outbreeding, genetic benefits of mating with bearers of elaborate sexually selected traits might be swamped by the costs of decreased fitness of female progeny due to sexually antagonistic effects.

Darwin developed his theory of sexual selection to explain the evolution of elaborate traits, such as deer antlers and peacock trains, that are likely to decrease survival but increase reproductive success (Darwin 1871), helping in male–male combat and in attracting the opposite sex (Andersson 1994; Andersson and Simmons 2006). Energetic costs of developing and maintaining these sexually selected traits in males imply that they can act as an honest signal of male genetic quality. Thus, female preferences for such traits can accrue indirect fitness benefits due to increased fitness of their progeny (Zahavi 1975; Andersson 1986; Grafen 1990; Houle and Kondrashov 2002). However, support for this “good genes” hypothesis is equivocal. While some studies that manipulated deleterious mutation load directly (Herdegen and Radwan 2015), exposed it via inbreeding (van Oosterhout et al. 2003) or inferred it from genomic data (Dugand et al. 2019) find support for sexually attractive males carrying lower mutation load (but see Prokop et al. 2010), a meta‐analysis found no significant association between male attractiveness and progeny fitness, except for increased sexual attractiveness of male progeny (Prokop et al. 2012). These apparently conflicting results may be reconciled if genes associated with the expression of sexually selected traits in males also have negative pleiotropic effects on other male fitness components, or on female fitness (Lande 1980; Rice and Chippindale 2001; Radwan et al. 2015; Zajitschek and Connallon 2018).

Indeed there is some evidence that sexual weapons or ornaments show negative genetic correlations with female fitness (Harano et al. 2010; Plesnar‐Bielak et al. 2014), as well as juvenile (Brooks 2000) and adult male survival (Johnston et al. 2013). Thus, even if males expressing the more elaborate sexual traits carry a lower load of deleterious mutations, their progeny, particularly of the opposite sex, may not necessarily have higher fitness. Empirical studies simultaneously quantifying both mutation load and negative pleiotropy are needed to better appreciate the relative contribution of each to male and female fitness. Here, we report such a study using inbred lines derived from males differing in the expression of a heritable sexually selected weapon.

Our model species for this study was the bulb mite *Rhizoglypus robini*, a male‐dimorphic species in which aggressive fighter males express a sexually selected weapon in the form of thickened and sharply terminated third pair of legs, which in scrambler males and in females are unmodified and resemble the other pairs. This weapon is costly to produce and condition‐dependent (Smallegange 2011b; Radwan 1995), therefore its significant heritability (Radwan 1995, 2003a; Smallegange and Coulson 2011) could potentially result from differences in male genetic quality, with the weapon expressed mostly by males with a lower load of deleterious mutations. Indeed, proportions of morphs among progeny do not conform to standard Mendelian segregation, although the contribution of major effect genes cannot be excluded (Radwan 1995). Consistent with this "good genes" mechanism, fighters were reported to have better survival and higher reproductive success than scramblers (Radwan and Klimas 2001). By contrast, there is some evidence that genes associated with fighter morph expression are detrimental when expressed in females. Females from artificial selection lines nearly fixed for fighter males were less fecund and shorter‐lived compared to females from lines nearly fixed for scrambler males (Plesnar‐Bielak et al. 2014). One explanation for this phenomenon would be that fighter males carry a higher load of deleterious mutations across their genome, but this would be inconsistent with condition‐dependent expression of the weapon. Alternatively, during mass selection on morph frequencies, natural and sexual selection could simultaneously act within selection lines. The resulting lower female fecundity in fighter‐selected lines could be due to sexually antagonistic alleles being favored by sexual or natural selection. Finally, if expression of the weapon is directly affected by genes unrelated to condition, such genes might be negatively associated with female fitness. For example, the genes for weapon expression could be located on inversions also carrying female‐harm genes. Here, we estimated both the load of deleterious mutations and gender load using inbred lines derived from females mated with either fighter or scrambler males (henceforth F‐ and S‐lines, respectively).

Because deleterious mutations segregating in populations are typically partially recessive, their deleterious effects are easiest to detect under inbreeding (Tomkins et al. 2004). Inbreeding increases genome‐wide homozygosity leading to inbreeding depression attributable mainly to the expression of a large number of small effect, partially recessive mutations (Charlesworth and Willis 2009). To test whether expression of the weapon is associated with a lower load of deleterious recessives, we compared inbreeding depression for female fecundity between F‐ and S‐lines. We chose female fecundity as our focal trait because, being typically highly polygenic, it can capture the load of deleterious mutations across a large proportion of the genome (Houle 1998; Radwan 2003b). To test for gender load associated with genes underlying fighter expression, we compared female fecundity between outbreds derived from F‐ and S‐lines.

## Methods

We collected mites from onions gathered in a field in Mosina, Wielkopolskie, Poland, in October 2017, and maintained them under laboratory conditions of 24°C at >95% humidity, on a diet of ad libitum powdered yeast. We established inbred lines from the first generation that hatched in the laboratory, and used the remaining individuals to found a stock culture. To obtain virgin females to start our inbred lines, we placed 100 larvae in individual vials (0.8 cm diameter glass vials with plaster base soaked with water) and after they reached adulthood, we set up males and females in single‐pair crosses. The proportion of both male morphs among our inbred lines reflected those found in the original population: we established 26 F‐lines and 15 S‐lines. In the following generations, we maintained these inbred lines by sib–sib mating as described below. Individuals were selected randomly for the next generation, except that we used males of the same morph as the founder males for each line, to help maintain differences in morph proportions between F‐ and S‐lines (Fig. S1). To do this, we reared 20 larvae through to adulthood for each line, and mated each male of the appropriate morph with a randomly selected virgin female, giving typically two to six replicate families per generation. One of these families was randomly selected each time to found the next generation. If the first selected family failed to produce offspring, another was randomly selected to replace it; this occurred on five separate occasions at generation 2 (one in S lines and four in F lines), three cases at generation 3 (all three in F lines), and seven at generation 4 (three in S lines and four in F lines). After four generations of sib–sib mating (inbreeding coefficient *F* = 0. 594) we estimated inbreeding depression and gender load in *F*‐ and *S*‐lines by comparing female fecundity between F4 inbred and outbred lines. We used 14 of the 15 available S‐lines (one line did not produce enough individuals) and a subset of 19 randomly selected F‐lines. We produced outbred females by setting up crosses between different inbred lines founded by the same male morph type. Through this method we generated 18 different combinations of S‐lines, and 17 combinations of F‐lines, with each inbred line used in no more than two such combinations. After females laid eggs, we randomly selected 10 larvae from each inbred and outbred family and from this selected two to four virgin females at maturity to mate with males from the stock culture. This allowed us to estimate inbreeding depression for female fecundity independently from male fertility. Males of both morphs were used as mates. We counted the number of eggs laid in a period of 7 days, during which females had continuous access to a male, in order to avoid confounding effects due to sperm limitation. Pairs were transferred to fresh vials once at 4 days after male introduction. Because female reproductive output remains high and changes little over the first 3 weeks (Tilszer et al. 2005), eggs laid over 7 days can be used as a proxy for female lifetime fecundity.

Data on female fecundity were analyzed by using a linear mixed model (LMM). Normality of error distributions was verified with diagnostic plots of residuals and Shapiro–Wilk tests. Treatment (inbred or outbred), founder male morph, their interaction, and morph of a male with which the focal female was paired, were entered as fixed factors. A significant founder × treatment interaction was expected if inbreeding load differed between lines founded by scramblers and fighters. Maternal and paternal line identities were entered as random factors. Random slopes were modeled to allow for lines to respond differentially to inbreeding. LMM was implemented by the *lmer* function in the *lme4* package (Bates et al. 2015), with *P*‐values calculated based on Satterthwate's approximations using the package *lmerTest* (Kuznetsova et al. 2014), implemented in R version 3.5.1 (R Core Team 2018).

## Results and Discussion

**Figure 1:**
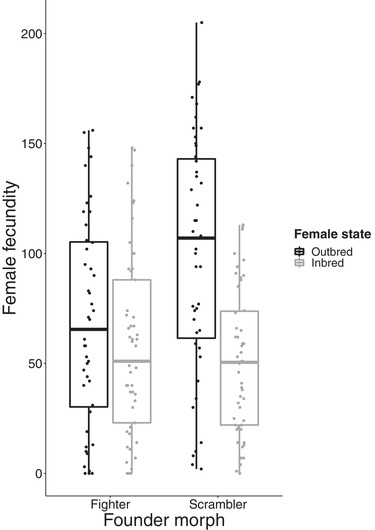
Effects of inbreeding and inbred lines founder morph on female fecundity. The box encloses values between the first and third quartiles of the data (the inter‐quartile range, IQR), while the horizontal bar within the box indicates the median. Whiskers extend from the box to the largest/smallest values that are within 1.5× the IQR of the box.

We found that female fecundity was significantly influenced by the interaction between founder morph and inbreeding (LMM: *t* = −3.096, *P* = 0.003). Female fecundity in inbreds dropped, compared to outbreds, more markedly in S‐lines than in F‐lines (Fig. 1, Table 1). This indicates that a higher load of deleterious recessives segregated in S‐lines compared to F‐lines. In addition to a lower mutation load in fighter founders, this result could be due to more effective purging of deleterious recessives in F‐lines. This could occur because fighter males selected as sires in each generation of inbreeding in these lines were likely in better condition compared to scrambler males (Radwan 1995; Smallegange 2011b). In any case, the apparent lower load of deleterious recessives in F‐lines did not result in higher fitness for outbred females. On the contrary, inspection of Figure 1 and the model summary (Table 1) shows that fecundity of outbred females derived from S‐lines was significantly higher than in F‐lines (the effect of scrambler founder compared to outbred fighter in intercept, *t* = 2.557, *P* = 0.019). This result may appear paradoxical, as inbreeding depression is thought to be caused mainly by the accumulation of mildly deleterious mutations (Charlesworth and Willis 2009), which are often only partially recessive (Crow and Simmons 1983; Barrett and Charlesworth 1991). Likewise in the bulb‐mites, there is evidence for partially recessive alleles of small effect having a major contribution to inbreeding depression, as estimated from female fecundity effects (Radwan 2003b; Jarzebowska and Radwan 2010). If this is true then the accumulation of recessive alleles across multiple genetic loci should have negatively affected female fecundity more in S‐lines that demonstrated greater evidence of inbreeding depression. The opposite pattern that we detected indicates that genes associated with expression of a costly weapon have a negative effect on female fitness, that is, cause gender load.

**Table 1:** Results of linear mixed model with female fecundity as a dependent variable, and the morph of inbred line founder (Founder morph), treatment (inbreeding vs. outbreeding) and morph of a male the female mated with (Mate morph) as predictors. The identities of inbred lines that the female and male came from were entered as crossed random factors

		Parameter estimate	Standard error	*df*	*T*	*P*‐value
Fixed effects	(Intercept)	75.44	9.67	26.34	7.80	<0.001
	Founder morph ‐ scrambler	32.69	12.79	20.28	2.557	0.019
	Treatment ‐ inbred	−13.12	9.93	51.75	−1.322	0.192
	Mate morph ‐ scrambler	−10.82	5.77	152.89	−1.874	0.063
	Founder morph* Treatment state	−40.95	13.23	53.47	−3.096	0.003
		Variance	Standard deviation			
	Maternal line:					
Random effects	(Intercept)	512.96	22.65			
	Treatment ‐ inbred	44.80	6.69			
	Paternal line:					
	(Intercept)	72.06	8.49			
	Treatment ‐ inbred	31.37	5.60			
Residual		1556.67	39.46			

Our results are consistent with another report of decreased female fecundity in bulb‐mites following near‐fixation of the male weapon phenotype in mass‐selection lines (Plesnar‐Bielak et al. 2014) and with results of a similar experiment on horned beetles (Harano et al. 2010). Interestingly, the effect sizes reported in these studies are highly comparable with those found here (15‐25%; here: 22% ± 8% decline in fecundity). While female harm associated with a sexually selected weapon in males appears to be a general phenomenon in *R. robini*, the results of the experiment by Plesnar‐Bielak et al. (2014) could be also explained by inadvertent selection for fighter‐ or scrambler‐beneficial alleles, with the former possibly having sexually antagonistic effects. Here, natural and sexual selection were minimized by using inbred lines established by sib‐mating of randomly selected, monogamous pairs, such that inadvertent selection on life histories was much reduced. Nevertheless, by selecting fighter males in F‐lines, we could have selected alleles that increased the probability of weapon expression, for example, those contributing to male phenotypic condition. This would imply that such genes have negative pleiotropic effects on female fitness. Alternatively, genes underlying morph expression could have pleiotropic effects on other sexually antagonistic traits expressed in both sexes (Hosken 2011), or be located in regions of low recombination (e.g., Küpper et al. 2016) containing linked genes that negatively impact female fitness.

Our results indicate that both mutation load and negative pleiotropy can contribute to the maintenance of genetic variance for male weapon expression in the bulb mite. Because morph expression in this species is condition‐dependent (Smallegange 2011a; Radwan 1995), deleterious mutation load should be negatively correlated to expression of sexual weapons, which is consistent with our evidence for a higher inbreeding load in S‐lines. The genetic variance may be further augmented by balancing selection arising from sexual antagonism and other forms of negative pleiotropy (Zajitschek and Connallon 2018). Our results highlight sexually antagonistic effects associated with elaboration of traits under sexual selection, and imply that such effects can swamp any genetic benefits of mating with high‐condition males. If our results reflect the general property of sexually selected traits, then sexual antagonism associated with their evolution may explain the absence of good genes benefits of mating with their bearers despite substantial heritability of these traits (Prokop et al. 2012; Prokuda and Roff 2014). Comparatively high gender load can also limit the potential of good genes sexual selection to improve population fitness, and interspecific differences in this load may help explain why elaborate sexual traits are associated with increased risk of extinction in some taxa (Martins et al. 2018) but with decreased risk in others (Parrett et al. 2019).

## DATA ARCHIVING

Data available from the Dryad Digital Repository: https://doi.org/10.5061/dryad.ghx3ffbkc

Associate Editor: L. Spurgin

Handling Editor: D. W. Hall

## Supporting information

Fig. S1. Proportions of fighter morph in fighter (black) and scrambler (grey) inbred lines across 4 generations of brother‐sister mating.
