# Supplementary material for: Sexually selected male weapon is associated with lower inbreeding load but higher sex load in the bulb mite
Source: Evolution. 2020 Jul 6;74(8):1851–5. doi: 10.1111/evo.14033 (PMC7496443; doi:10.1111/evo.14033)
Supplement: Supplementary file 1 — Fig. S1. Proportions of fighter morph in fighter (black) and scrambler (grey) inbred lines across 4 generations of brother‐sister mating. [file EVO-74-1851-s001.docx]

Title: Sexually-selected male weapon is associated with lower inbreeding load but higher gender load in the bulb mite

Fig. S1. Proportions of fighter morph in fighter (black) and scrambler (grey) inbred lines across 4 generations of brother-sister mating.
